# Coupling of chromatography with surface-enhanced Raman spectroscopy: trends and prospects

**DOI:** 10.3389/fchem.2025.1548364

**Published:** 2025-02-26

**Authors:** Ekaterina V. Dmitrieva, Olesya O. Kapitanova, Shixian Lv, Oleg G. Sinyashin, Irina A. Veselova

**Affiliations:** ^1^ Faculty of Material Sciences, Shenzhen MSU-BIT University, Shenzhen, China; ^2^ Chemistry Department, Lomonosov Moscow State University, Moscow, Russia; ^3^ School of Materials Science and Engineering, Peking University, Beijing, China; ^4^ Federal Research Center Kazan Scientific Center of Russian Academy of Sciences, Kazan, Russia

**Keywords:** surface-enhanced Raman spectroscopy, chromatography, TLC-SERS, HPLC-SERS, method development

## Abstract

Surface-enhanced Raman spectroscopy is a powerful analytical technique for the determination of analytes with the advantages of sensitivity, portability, and simplicity, able to provide structural information for the identification of compounds. However, when it comes to the analysis of complex samples, matrix components may interfere with the analyte quantification. To overcome this shortcoming, a number of approaches have been proposed, such as extraction techniques. Among them, the coupling of chromatography with surface-enhanced Raman spectroscopy seems to be promising. It allows combining the advantages of both techniques, i.e., high efficiency of chromatographic separation and high sensitivity of surface enhanced Raman scattering detection, and makes possible simultaneous quantification of multiple analytes. The review summarizes the latest achievements in the combination of these techniques.

## 1 Introduction

Despite the rapid development of analytical methods, most of them still suffer from insufficient sensitivity and selectivity, high cost, and inapplicability for on-site analysis. Therefore, there exists a need to develop techniques devoid of these shortcomings. One such technique is surface enhanced Raman scattering (SERS), discovered in the 1970s (Ay et al., 2024; Jeanmaire and Van Duyne, 1977; Wang et al., 2022), with the advantages of portability, high sensitivity, rapid and powerful non-destructive detection (Han et al., 2023; Zhang et al., 2017), which is very sensitive to both chemical and biological species. It is a molecular vibration spectroscopy technique, resulting from an inelastic scattering process, able to provide structural information of a molecule (Zhang et al., 2017). Two mechanisms of signal enhancement in SERS have been proposed, namely, electromagnetic enhancement and charge-transfer or chemical enhancement (Han et al., 2023; McNay et al., 2011; Zhang et al., 2017). Chemical enhancement is generally limited to two orders of magnitude, while electromagnetic–up to 14 orders of magnitude (Han et al., 2023; Zhang et al., 2017). Historically, these two mechanisms were thought to be quite different (McNay et al., 2011). To date, numerous studies have revealed that these two mechanisms work together; however, their contribution varies (Han et al., 2023; Jensen et al., 2008). For both mechanisms, the analyte should be first adsorbed on a SERS active substrate followed by substrate irradiation by monochromatic radiation usually from a laser (McNay et al., 2011).

Electromagnetic enhancement is caused by localized surface plasmon resonance (LSPR) of compounds at the nanostructured surface of noble metals, e.g., Ag, Au, or bi/trimetallic noble metal alloys (Duy Vu et al., 2024), and depends on the roughness of the metal surface (Han et al., 2023). For the enhancement to occur, the molecule and surface of substrate should be in very close vicinity, i.e., less than 10 nm (Freye et al., 2013; Gao et al., 2015). Chemical enhancement is considered to involve electron transfer between the analyte and nanostructured surface with the formation of a charge transfer complex (Gao et al., 2015; McNay et al., 2011), which alters the molecule polarization (Ay et al., 2024; Duy Vu et al., 2024; Zhang et al., 2017). The morphology and size of the substrates play an essential role in the enhancement of electromagnetic field (Sha et al., 2022). Even slight variations of such parameters of colloidal solutions as cluster sizes and shapes produce changes in the enhancement factors by several orders of magnitude. Optimum enhancement can be observed for metallic substrates with the diameters between 10 and 150 nm (Sackmann and Materny, 2006). To achieve better performance, structures with different shapes, e.g., nanospheres, nanorods, nanostars, etc., were synthesized (Sha et al., 2022). In practice, a substrate should provide reasonable enhancement, be reproducible, stable, and robust (Freye et al., 2013).

SERS is highly efficient in the analysis of samples with a simple matrix. However, it is not a separation technique (Zhang et al., 2017), and when it comes to real sample analysis, matrix components may interfere with the determination of target analytes (Dai and He, 2023; Han et al., 2023; Lee et al., 2018; Minh et al., 2019), e.g., due to high fluorescence of natural compounds or interactions with irrelevant components present in the samples (Minh et al., 2023; Minh et al., 2024; Zhang et al., 2017). The most apparent solution of this issue is a preliminary separation of a complex mixture. For this, several approaches can be applied, such as liquid-liquid extraction (Alves et al., 2020), solid-phase extraction (Radu et al., 2016), dispersive liquid-liquid extraction (Santhoshkumar et al., 2025), cloud-point extraction (Zhang et al., 2021), or utilization of filter membranes (Fateixa et al., 2018; Qu et al., 2017; Yu and White, 2012; Wigginton and Vikesland, 2010). These approaches provide analyte purification from matrix components along with its concentration. However, analyte losses during extraction can occur, and these techniques can be applied for a limited number of compounds. For the simultaneous determination of multiple analytes, chromatography is the most powerful method. This method is suitable for analysis of complex samples and allows the separation of analytes into narrow zones depending on their physicochemical properties. One of the most informative detection methods coupled with chromatography is mass spectrometry, which allows identification of molecules based on their mass-to-charge ratios (m/z) and fragmentation patterns. However, the instruments are expensive, hardly portable, and require highly qualified personnel. At the same time, surface-enhanced Raman spectroscopy, capable of analytes identification based on their fingerprint spectra (Wang et al., 2021), is devoid of these shortcomings. In this regard, hyphenation of Raman spectroscopy with chromatography has been proposed and is widely applied nowadays. The review summarizes the results of studies utilizing chromatographic separation with subsequent SERS detection for the analysis of real samples and discusses future perspectives of the method.

## 2 Combination of surface-enhanced Raman spectroscopy with chromatography

**FIGURE 1:**
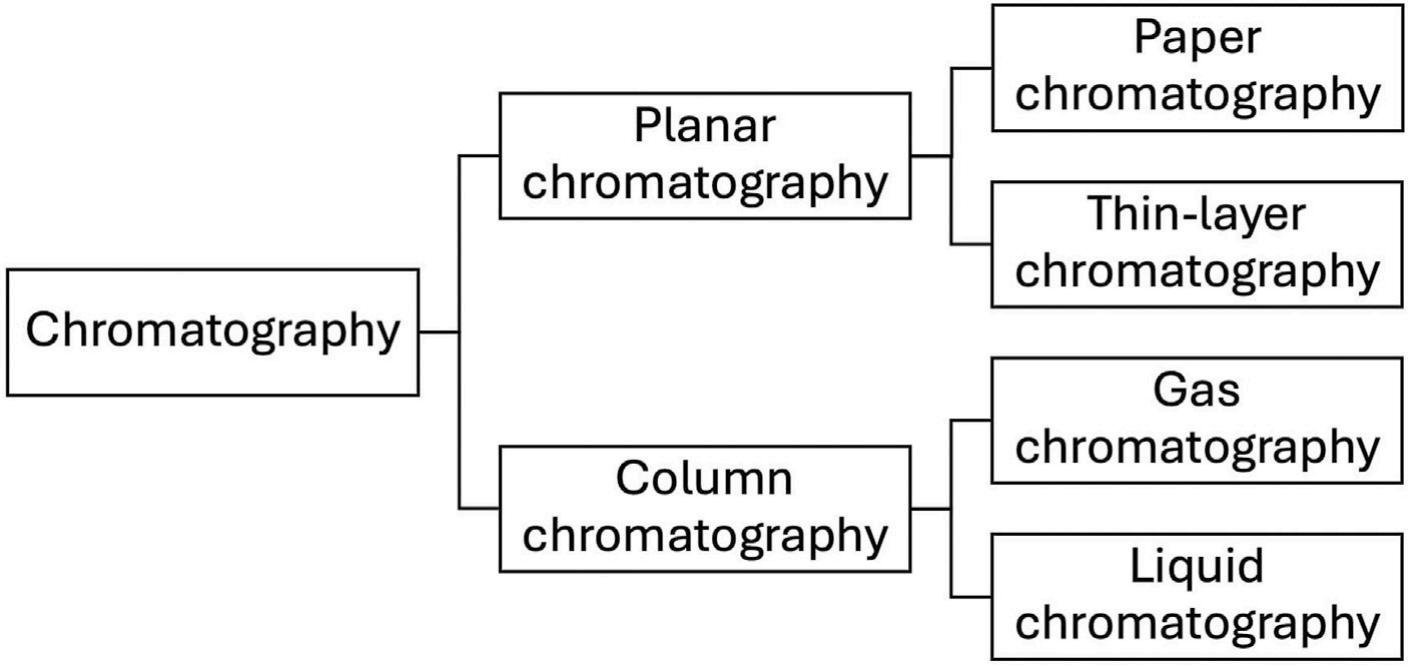
Classification of chromatographic methods coupled with SERS detection.

Chromatography is based on the analyte distribution between two phases, i.e., mobile and stationary, followed by detection of separated analytes applying various physicochemical principles. The chromatographic methods are divided into planar and column. Paper chromatography and thin-layer chromatography (TLC) are related to planar chromatography. In these methods, the separation of analytes is carried out on plane surfaces and is driven by capillary forces. In gas chromatography (GC) and liquid chromatography (LC), the separation is conducted in columns. The schematic of this classification is given in Figure 1. Nowadays, all these methods were combined with surface-enhanced Raman spectroscopy. Among them, the coupling of SERS with planar chromatography is easier to implement; as a result, it has been applied more widely.

### 2.1 Planar chromatography

#### 2.1.1 Thin-layer chromatography

Compared to column chromatography, thin-layer chromatography with SERS detection has the advantages of rapid on-site detection with low cost, decreased sample pretreatment, and ability to simultaneously analyze several samples (Zhu et al., 2019). In thin layer chromatography, chromatographic plates with a sorbent layer are utilized as the stationary phase. After spiking the sample at the starting line, the plates are placed in a separation chamber containing the mobile phase for the development. The analytes with the eluent pass through the sorbent by capillary forces and are separated on the plate according to their affinity to the stationary phase. The parameter used for analyte identification is the retention factor (Rf), i.e., the ratio of the distance passed by the analyte to the distance passed by the eluent (solvent front). After compounds separation, the analytes can be detected by means of several methods, for example, densitometry or fluorescence spectroscopy. TLC provides fast and efficient analyte separation, but the detection methods lack sensitivity and selectivity; hence, SERS was proposed as an alternative due to their high compatibility (Han et al., 2023). The combination of TLC and SERS was first described in 1977 (Henzel, 1977; Pozzi et al., 2012) and nowadays has found numerous applications in various fields from food safety to environmental protection. Other essential advantages of the method include the lack of cross-contamination due to the use of disposable TLC plates, ability to simultaneously analyze several samples, and its portability. As a result, it can be used for on-site analysis of complex samples. Another interesting application of TLC-SERS is on-site quantitative monitoring of chemical reactions. The reaction mixture is spiked on a TLC plate; after the plate development, SERS is applied for detection, which enables to distinguish even incompletely separated compounds according to their characteristic Raman bands without loss of chemicals. In addition, it can be applied to the detection of compounds unstable under ultraviolet radiation and compounds present at low levels (Chen et al., 2015a; Fukunaga et al., 2023; Zhang et al., 2014).

**FIGURE 2:**
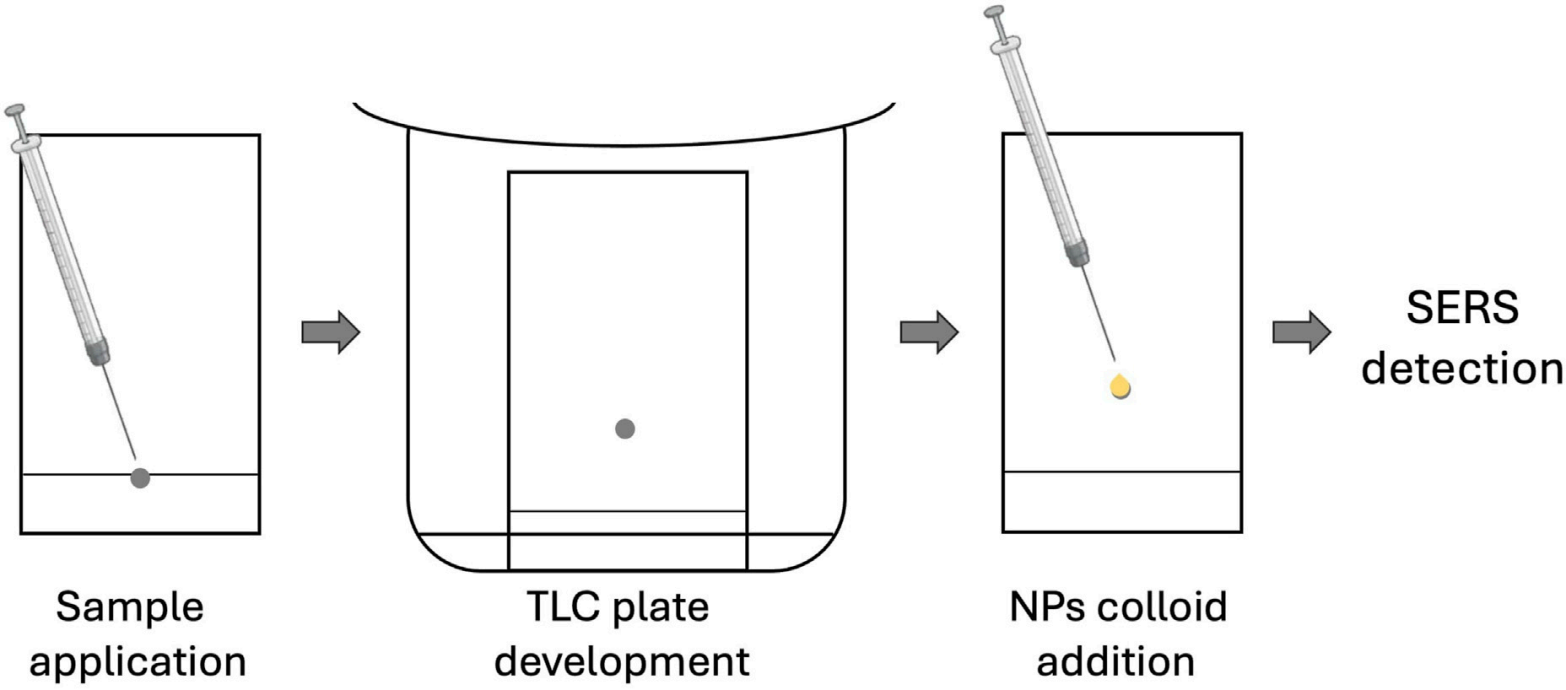
Schematic of TLC-SERS utilizing conventional TLC plates.

**FIGURE 3:**
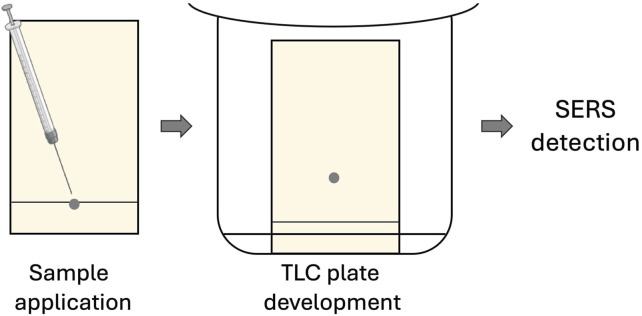
Schematic of TLC-SERS utilizing modified TLC plates.

There exist several approaches for TLC-SERS assay. The first one is based on the analyte separation on commercially available TLC plates with conventional stationary phases followed by the addition of metal nanoparticle (NP) colloids on the developed plate for the sensitive SERS detection (Figure 2); these substrates have random morphology (Freye et al., 2013; Zhao et al., 2019). The other is based on the stationary phase modification to obtain a SERS-active surface with subsequent analyte separation and detection (Figure 3) (Chen et al., 2012; Han et al., 2023; Minh et al., 2019); the substrates have deterministic morphology and were termed as engineered substrates (Freye et al., 2013).

In the former approach, commercially available TLC plates are mostly used to separate target analytes. Nowadays, there exists a wide range of TLC plates allowing to separate analytes with various physicochemical properties. Even though TLC plates are generally weak Raman scatters (Cimpoiu et al., 2005; Orinak et al., 2008), several studies have revealed that they are able to provide pronounced background Raman scattering from the stationary phase (Horváth et al., 2000; István et al., 2003; Zhu et al., 2014), which should be considered during optimization of conditions. Among the stationary phases, the most commonly used is silica gel. Although it does not provide significant background to the SERS signal, the interactions between the Si−OH groups of the TLC plate and the analyte can result in hydrogen bonding leading to a shift in the obtained spectrum (Freye et al., 2013; White, 1985). What is more, the solvents for the mobile phase should be carefully selected; for example, acetonitrile was reported to adsorb readily to the silver surface and disturb SERS measurements at low analyte concentrations (Trachta et al., 2004). To achieve the signal enhancement by SERS detection, valuable metal colloids are introduced to the separated spots of analytes. The spots of analytes can be easily visualized, since most TLC plates contain a fluorescent indicator. To obtain a strong SERS signal, the choice of NPs material is crucial (Yao et al., 2013). For obtaining the best results, nanoparticles preparation procedure, concentration, and spiking volume are optimized (Orinak et al., 2008; Yao et al., 2013). The most used metals for the preparation of colloid solutions are gold and silver due to the simplicity of their preparation by a chemical reduction method, relatively low cost, reasonable stability, and narrow particle size distribution (Han et al., 2023; McNay et al., 2011). Even though gold is more inert in comparison to silver, it quite strongly absorbs at around 500 nm. Therefore, silver is more suitable in this wavelength range. At the same time, silver has higher chemical reactivity reducing it lifetime (McNay et al., 2011; Murugan et al., 2021).

Application of conventional TLC plates with subsequent addition of noble metal colloids for SERS detection has been described in various fields, i.e., dietary supplements analysis, food quality control, analysis of environmental samples, or showing the method capability on model solutions (Table 1), for the quantification of antitussive and antiasthmatic drugs (Fang et al., 2016), antidiabetic drugs (Minh et al., 2023; Zhu et al., 2014), lipid-lowering agents (Zhu et al., 2017), β-carboline alkaloids (Pozzi et al., 2012), aflatoxins (Qu et al., 2018), pesticides (Kang et al., 2019; Yao et al., 2013), substituted aromatic pollutants (Li et al., 2011), synthetic dyes (Cañamares et al., 2014; Ferretti et al., 2024), etc. Solid samples were mostly prepared for analysis by homogenization followed by their extraction, centrifugation, and spotting supernatant on the starting line of a TLC plate for separation. Liquid samples were diluted with the solvent and centrifuged to reduce matrix effects. In addition to introducing noble metal colloids after TLC separation, immersing TLC plate in silver ion solution was proposed. This approach was applied to the assay of binary mixtures of cresyl violet, bixine, crystal violet, and Cu(II) complex of 4-(2-pyridylazo)resorcinol. Prior to analyte separation, a silica gel 60 TLC plate was immersed in silver nitrate solution. Then, the separated analytes were detected by SERS (Herman et al., 2013). Another implementation of TLC-SERS combination was shown in (Li et al., 2021) for the quantification of 14 citrus flavonoids in various matrices. After their separation on a normal-phase TLC plate, the analytes from spots were extracted in methanol. The supernatant was then analyzed by SERS. A similar approach was applied to the quantification of cotinine and trans-3′-hydroxycotinine, metabolites of nicotine and indicators of smoking status, in urine (Huang et al., 2013). However, non-uniform adsorption of metal colloids on the stationary phase owing to its porosity may occur, and the process of dispensing colloids may disrupt the separated target molecules due to the spot partial solubilization thus affecting accuracy and reproducibility of results (Dai and He, 2023; Han et al., 2023; Sciutto et al., 2017; Takei et al., 2015; Zhao et al., 2019). The phenomenon of the formation of a ring-shape spot after the addition of nanoparticles caused by their aggregation was termed as the coffee ring effect (CRE): single CRE if nanoparticles aggregate alone and double CRE if nanoparticles aggregate with the analyte (Minh et al., 2023). The migration of the nanoparticles and analyte due to the CRE can also affect the repeatability of results (Yao et al., 2022). Another important observation is based on the time of SERS signal measurement since Ag or Au colloid spiking to the analyte spot. The nanoparticles were reported to generate hot spots during solvent volatilization, and solvent volatilization-induced analyte concentration occurred. The SERS signal was the strongest when the droplet tended to become dry. However, the reproducibility of the results was poor even if continuous measurements during solvent evaporation were carried out (Kang et al., 2019). The signal enhancement through modification of the TLC plate surface by adding a thin film of water was also noted in (István et al., 2003; Szabo and Winefordner, 1997). To prevent local drying effects from the laser, a cover glass was used (István et al., 2003). The control of nanoparticles colloid solution stability is also quite challenging, since they are prone to spontaneous aggregation, which may also affect reproducibility of results (Han et al., 2023; Lee et al., 1998).

**TABLE 1:** TLC-SERS using conventional TLC plates.

Matrix	Analyte	Sample preparation	TLC		SERS		LOD	References
			Stationary phase	Mobile phase	SERS-active species	Detection		
Botanical dietary supplements	Gemfibrozil, benproperine phosphate, chlorphenamine maleate	Ultrasound-assisted extraction with methanol	High performance silica gel 60-F254	Dichloromethane: methanol: water (9 : 1: 0.1, v/v)	DMF and silver colloid (2 : 1, w/w)	785 nm laser source; ×20 objective; 220 mW excitation power; 15 s	0.001–0.01 μg/μL	Zhu et al. (2019)
Botanical dietary supplements	Diphenhydramine hydrochloride, benproperine phosphate, chlorphenamine maleate, phenformin hydrochloride	Ultrasound-assisted extraction with methanol	High-performance silica gel 60-F254 (glass back plate)	Dichloromethane: methanol: water (9 : 1: 0.1, v/v)	50% glycerol silver colloid	785 nm excitation wavelength; 5 cm^−1^ resolution; ×20 objective; 200 mW excitation power; 5 s	0.005–0.01 μg/μL	Fang et al. (2016)
Botanical dietary supplements	Phenformin hydrochloride, metformin hydrochloride, rosiglitazone maleate, pioglitazone hydrochloride	Ultrasound-assisted extraction with methanol	Silica gel	Dichloromethane: methanol: water (8 : 2: 0.2, v/v)	Silver colloid	785 nm excitation wavelength; 5 cm^−1^ resolution; ×20 objective; 100 mW laser power; 5 s	0.001%–0.2%	Zhu et al. (2014)
Herbal products (herbal extract, granule, and capsules)	Sildenafil	Vortex- and ultrasound-assisted extraction with methanol	Silica gel 60 F254	Ethyl acetate: isopropanol: 25% ammonia (45 : 5: 2.6, v/v)	Silver colloid	633 nm excitation wavelength; ×50 objective; 60 s	2 ng/spot	Minh et al. (2019)
Herbal products (capsules, pills, concentrated extract)	Sildenafil	Ultrasound-assisted extraction with methanol	HPTLC Silica gel 60 F254 (aluminum base)	Ethyl acetate: isopropanol: 25% ammonia solution (45 : 5: 1, v/v)	Nano silver colloid	633 nm excitation wavelength; ×50 objective; 60 s	1.65 ng/spot	Minh et al. (2019)
Herbal products	Glibenclamide, metformin	Vortex- and ultrasound-assisted extraction with methanol	Silica gel 60 F254	n-Butyl acetate: methanol: formic acid (11 : 2.5 : 1.5, v/v)	Silver colloid	633 nm excitation wavelength	0.5–1 μg/spot	Minh et al. (2019)
Herbal dietary supplements	Nicotinic acid, simvastatin, probucol, fenofibrate, bezafibrate, ciprofibrate, atorvastatin calcium, fluvastatin sodium, pravastatin tetramethylbutylamine	Ultrasound-assisted extraction with methanol	Silica gel 60-F254 (aluminum back plate)	Petroleum ether: acetic ether: acetic acid (5.5 : 2.8 : 1, v/v)	Silver colloid	785 nm excitation wavelength; 5 cm^−1^ resolution; ×20 objective; 200 mW laser power; 15 s	0.002%–0.10%	Zhu et al. (2017)
Tea leaves	Dursban, phoxim, parathion, methidathion, isocarbophos	Extraction with ethyl acetate and acetone	Slica 80-GF254 TLC with fluorescent indicator	Petroleum ether: ethyl acetate (9 : 1, v/v)	Silver colloid	785 nm excitation wavelength; ×20 objective; 24 mW laser power	0.1 ppm (LOQ)	Yao et al. (2013)
Seeds of Syrian rue (*Peganum harmala*)	Harmalol, harmaline, harmane, harmine	Extraction with methanol	Silica gel	Trichloromethane: methanol: 10% NH_3_ (80 : 20: 1.5, v/v)	Silver colloid and 0.5 M KNO_3_ aqueous solution	488 nm excitation wavelength; ×20 objective; 30 s	-	Pozzi et al. (2012)
Milk	Melamine	Dilution with methanol	Silica gel 60	Acetone: chloroform: ammonia (7 : 0.5 : 2, v/v)	Gold nanoparticles	785 nm excitation wavelength; 2 cm^−1^ resolution; 30 mW laser power; 5,000 ms	-	Zhao et al. (2019)
Hot pot condiments	Papaverine, noscapine	QuEChERS	-	Methylbenzene: acetone: ethanol: ammonium hydroxide 20 : 20: 5 : 1 (v/v)	Silver colloid	785 nm excitation wavelength; ×50 objective; 50 mW laser power; 5 s	0.5–3 mg/kg	Hu et al. (2017)
Fish	Histamine	Extraction with 10% trichloroacetic acid aqueous solution	Silica gel 60	Methanol: acetone: ammonia (25%), (3 : 7: 0.5, v/v)	Silver nanoparticles and 2 M NaCl aqueous solution	633 nm excitation wavelength; 10 s	-	Xie et al. (2017)
Pork	Metronidazole, tinidazole, ronidazole, nitrobenzimidazole, ornidazole, secnidazole, dimetridazole, 2-methyl-5-nitroimidazole, 2-nitroimidazole, 4-nitroimidazole, ipronidazole, metronidazole-OH, hydroxy dimetridazole and 5-chloro-1-methyl-4-nitroimidazole	Extraction with ethyl acetate and n-hexane	-	Methanol: ether: chloroform	Gold glue	785 nm excitation wavelength	0.1–100 mg/L	Shi et al. (2022)
Cherry	Phosmet, thiabendazole, triazophos	Ultrasound-assisted extraction with methanol and dichloromethane mixture (1:1, v/v)	Silica gel 60-F254	Dichloromethane: petroleum benzin: isopropanol (80 : 20: 3, v/v)	Silver nanoparticles and polyurethane mixture	785 nm excitation wavelength; 3 mW laser power; ×50 objective	0.02–0.8 μg/mL	Kang et al. (2019)
Peanuts	Aflatoxin B1, B2, G1, G2	Extraction with methanol	Silica gel 60-F254	Acetone: chloroform (9 : 95, v/v)	Gold colloid	785 nm excitation wavelength; 2 cm^−1^ resolution; 30 mW laser power; 10 s	6.0⋅10^−7^–1.1⋅10^–5^ M	Qu et al. (2018)
Human blood plasma	Apomorphine hydrochloride	EDTA addition	Silica	-	Silver colloid	785 nm excitation wavelength; ×10 and ×50 objective	10^–4^ M	Lucotti et al. (2012)
Wastewater	Aniline, benzidine, pyrocatechol, chlorobenzene (p-toluidine, p-nitroaniline, and lentine – library identification))	-	Silica 60-F254	n-Hexane: ethyl acetate (3 : 1, v/v)	Silver colloid and 20 mM NaCl mixture	785 nm excitation wavelength; 5 cm^−1^ resolution; 40 mW laser power; 50 s	0.008–0.2 ppm	Li et al. (2011)
Food contact materials (paper cups, polypropylene food containers, and polyethylene glycol terephthalate bottles)	Benzidine, 4-aminobiphenyl	Extraction with water, acetic acid, and ethanol	H254TLC	Petroleum ether: ethyl acetate (75 : 25, v/v)	Concentrated gold nanoparticle doped metal-organic framework (AuNPs/MIL-101(Cr))	785 nm excitation wavelength; 10 s	0.21–0.23 μg/L	Cai et al. (2020)
-	Rhodamine 6G, 4-aminothiolphenol, malachite green isothiocyanate	-	TLC C-18 silica gel	Ethanol	Silver nanocomposite	488 nm excitation wavelength; ×10 objective; 1.0 mW laser power; 4 s	1.47⋅10^−7^–2.74⋅10^–6^ M	Freye et al. (2013)
-	Purine, benzoic acid, 1-nitropyrene	-	Silica gel 60 HPTLC	-	Silver colloidal spheres	514.5 nm excitation wavelength; 10 mW laser power	-	Koglin, (1988)
-	Abietic acid, gibberellic acid, kaurenoic acid	-	Si60 F254s RAMAN	-	Silver colloid	514.5 nm excitation wavelength; ×40 objective	1.039 ng (gibberellic acid)	Orinak et al. (2008)

Abbreviations: LOD, limit of detection; LOQ, limit of quantification.

The latter strategy consists in the development of TLC plates with a built-in SERS structure (Table 2). This allows to avoid uneven distribution of metal nanoparticles on the TLC surface and obtain desirable SERS signal. The method was applied for the quantification of polyaromatic hydrocarbons (Wang et al., 2022), pesticides (Fang et al., 2024; Shen et al., 2021), antibiotics (Lu et al., 2024), synthetic dyes (Sciutto et al., 2017), and other compounds. In most cases, sample preparation was simple and fast, which is an apparent advantage of TLC. However, sophisticated equipment is required to prepare such substrates resulting in their high cost (Dai and He, 2023; Yao et al., 2022). Silver nanorods have been described as efficient substrates for TLC-SERS experiments. At the same time, their efficiency for the separation of different compounds is to be studied (Freye et al., 2013). Also, metal-organic frameworks (MOF) decorated with metal nanoparticles were proposed as SERS substrates due to their high ability to adsorb compounds owing to porous structure and ultra-high surface area. These structures are also not prone to external interference and undesirable aggregation, but the attempts to provide the metallic structures with stable NPs location and orientation failed. The strength of the SERS signal was found to be inversely dependent on the distance from the noble metal surface, making the thickness of the MOF a key element for optimization during the development of SERS substrates (Jiang et al., 2024). Alternatively, the fabrication of chips has been described (Chen et al., 2015b) for simple and fast SERS detection of polycyclic aromatic hydrocarbons in cooking oil.

**TABLE 2:** TLC-SERS using modified TLC plates.

Matrix	Analyte	Sample preparation	TLC conditions		SERS conditions		LOD	References
			Stationary phase	Mobile phase	SERS-active species	Detection		
Edible oil	Pyrene	-	Diatomite	Chloroform: n-hexane (1 : 6, v/v)	Gold colloid	785 nm excitation wavelength; 30 mW excitation power; 2 s	10^–4^ ppb	Wang et al. (2022)
Chili sauce	Sudan I	Dilution with acetone	Diatomaceous earth plates for TLC	Cyclohexane: ethyl acetate (6 : 1, v/v)	Gold nanoparticles	785 nm excitation wavelength; ×50 objective	0.5 ng/spot	Kong et al. (2017)
Chili oil		-						
Orange juice and kale leaves	Carbendazim	-	Diatomite TLC chips fabricated by spin coating	Chloroform: methanol (9 : 1, v/v)	Silver nanoparticles	785 nm excitation wavelength; 5 cm^−1^ resolution; 2 s	2 ppm	Shen et al. (2021)
Tuna	Histamine	Ultrasound-assisted extraction with 10% trichloroacetic acid	Diatomaceous earth TLC-SERS plates fabricated by spin coating on glass slides	Ethanol: ammonia (3 : 1, v/v)	Gold nanoparticles	785 nm excitation wavelength; 30 mW excitation power; 2 cm^−1^ resolution; 5,000 ms	10 ppm	Tan et al. (2019)
Beef	Ofloxacin	Centrifugation	Diatomite TLC plate	Chloroform: methanol: ammonia water (20 : 10: 3, v/v)	Silver colloid	785 nm excitation wavelength; 2 s	-	Lu et al. (2024)
-	Histamine, melamine, malachite green, rhodamine 6G, and sudan I	-	Diatomite biosilica channel arrays on a glass plate with integrated high density silver nanoparticles	Methanol: ethyl acetate: ammonia (6 : 2: 2, v/v)		10 mW excitation power; 1 s	-	Hou et al. (2022)
Milk	Melamine	Protein precipitation	Si nanowire arrays functionalized with silver nano-dendrites	Acetic acid	-	785 nm excitation wavelength; 200 mW excitation power; 1 s	2.5 ppm	Lee et al. (2018)
-	Methyl orange, cresol red, trans-1,2-bis(4-pyridyl)ethylene, methylene violet 2B	-	Ag nanorods UTLC plate	Methanol: acetonitrile		785 nm excitation wavelength; 30 mW excitation power; 1–10 s	10^−6^–10^−5^ M	Chen et al. (2012)
	Melamine, rhodamine 6G			Methanol				
-	Rhodamine 6G, crystal violet, and 1,2-di (4-pyridyl)ethylene	-	TLC plate with a built-in Ag nanoparticles layer; silica gel separation layer	Methanol: water (80 : 20)		633 nm excitation wavelength	-	Takei et al. (2015)
Skim milk	Melamine	Dilution with deionized water		Methanol				
Dyed wool	Synthetic dyes (Diamond Green, Methyl violet, Crystal violet, Rhodamine B, Rhodamine 6G, Eosin A, Martius yellow, Cochineal red A, Fast red AV, Indigo carmine, Methylen blue)	Extraction with methanol	A thin layer of AgI deposited on a gold coated glass slide (AgI@Au)	Ethanol: ammonia (9 : 1, v/v)	-	785 nm excitation wavelength; 10 mW excitation power; ×20 objective; 3 s	-	Sciutto et al. (2017)
Chili oil	Rhodamine B and sudan I	Extraction with methanol	GF254 TLC plate modified with Au@Ag NCs	Cyclohexane: ethyl acetate (6 : 1, v/v)		785 nm excitation wavelength; 80 mW excitation power; 5 s	1 ppm	Sha et al. (2022)
Soil aqueous solution	Thiram, triadimefon, benzimidazole, thiamethoxam	-	Self-assembly of Ag nanoparticles on TLC plate to preparate TLC-Ag SERS substrate	Hexane: methanol water: ethyl acetate (1 : 2: 4 : 2, v/v)	-	785 nm excitation wavelength; 1 cm^-1^ resolution; 30 mW excitation power; 5 s	-	Fang et al. (2024)
-	Rhodamine B, sudan III, malachite green, congo red	-	TLC fabric with a Cot@Au NPs@β-CD built-in SERS structure	Dichloromethane: methanol (3 : 1, v/v)		785 nm excitation wavelength; 0.5% excitation power; 10 s	-	Yao et al. (2022)
*Bellamya aeruginosa*	Hypertoxic natural toxin (microcystin-leucine-arginine)	-	ZIF-67/Ag NPs/Au NWs substrate implanted TLC plate	n-Hexane: ethyl acetate: acetonitrile: glacial acetic acid (1 : 3: 9 : 0.1, v/v)		532 nm excitation wavelength; 1 mW excitation power; 10 s	2.27⋅10^–9^ mM	Jiang et al. (2024)
Paprika powder	Sudan I	Ultrasound-assisted extraction with acetonitrile	Glass plate modified by MIPs	Hexane: chloroform (1 : 1, v/v)	Gold colloid	785 nm excitation wavelength; 25 mW excitation power; ×50 objective	1 ppm	Gao et al. (2015)

To improve the results of quantification by TLC-SERS, machine learning algorithms were also applied, i.e., principal component analysis (PCA)-back propagation neural network (Lu et al., 2024) and PCA with machine learning analysis based on support vector regression (SVR) (Tan et al., 2019), which resulted in elimination of interferences from complex samples.

#### 2.1.2 Paper chromatography

Paper chromatography is a type of planar chromatography utilizing a cellulose paper as the stationary phase. Despite its simplicity, low cost, and portability, the separation process is often slower, less accurate, and spots tend to diffuse higher in comparison to TLC (Wilson, 2000). Therefore, it is less commonly used than TLC. The combination of paper chromatography with SERS detection was applied for the quantification of dyes, i.e., crystal violet, malachite green, and basic fuchsin (Tran, 1984). A conventional chromatographic paper was utilized to separate the analytes, and the developed chromatograms were sprayed with silver colloidal hydrosols prior to SERS detection. Another approach consisted in the paper strip modification for the quantification of rhodamine-6G. For this, a paper strip was fabricated with Au nanodendrite on nickel foam structure (Duy Vu et al., 2024). Also, an inkjet-printed SERS substrate was fabricated by using paper and polymer membranes for sample cleanup and analyte separation with SERS detection. This approach was applied for the determination of melamine (Yu and White, 2013).

### 2.2 Column chromatography

#### 2.2.1 Liquid chromatography

In the analysis of complex samples containing compounds with similar structures or polarities, efficiency of TLC separation can be insufficient. Column chromatography, especially, high-performance liquid chromatography, has a significantly higher potential for the resolution of complex samples. Despite this, unique identification of compound peaks is still challenging [Trachta et al., 2004; Zhang et al., 2017). Coupling of HPLC with mass spectrometry significantly improved the capabilities of both methods and allowed to overcome this limitation. However, these instruments are expensive and require highly qualified personnel. SERS can be a suitable alternative allowing sensitive detection of analytes, because chromatographic separation of complex samples allows producing individual SERS signals (Zhang et al., 2017). The combination of HPLC and SERS was first described in 1988 for the determination of pararosaniline hydrochloride, an organic dye (Freeman et al., 1988; Khrushchev et al., 2022). In this method, an effluent from the chromatograph was mixed with Ag sol in a post-column mixing coil followed by SERS detection. Since that time, several studies utilizing HPLC-SERS detection have been reported, which can be divided into at-line and on-line modes. In the at-line approach, after the separation of analytes in the analytical column, the eluate fractions are collected for SERS analysis (Cowcher et al., 2014; Khrushchev et al., 2022; Seifar et al., 1999). This approach is easier to implement, and SERS conditions can be optimized for each fraction independently (Cowcher et al., 2014); however, the analysis time is increased in comparison to on-line approach. In contrast, the on-line approach requires the mixing unit of the eluate with nanoparticles solution and its passing through a Raman flow cell (Cabalin et al., 1993; Freeman et al., 1988; Sheng et al., 1991), but it has a disadvantage that a continued supply of nanoparticles is required (Goodacre et al., 2018). Alternatively, a capillary with SERS-active substrates can be connected to the chromatograph for on-line analysis (Nguyen and Schultz, 2016; Wang et al., 2015). However, such approach can be prone to memory effects, i.e., a subsequent analyte signal is disturbed by a previous compound residue (Cowcher et al., 2014; Zhang et al., 2017). Mobile phase composition can also cause Raman spectral interferences and affect SERS activity of the sol (Carrillo-Carrión et al., 2012). In this way, acetonitrile was noted to increase a background spectrum, therefore it is often replaced by methanol to increase sensitivity due to the reduced background signal (Trachta et al., 2004; Zhang et al., 2017).

For the sensitive determination of purine bases, both at-line and on-line approaches were applied. The former approach was implemented for the quantification of main purine and pyrimidine bases (adenine, guanine, thymine, and cytosine) and two of their most common degradation products (xanthine and hypoxanthine) using a novel SERS substrate based on ZnS/CdSe silver quantum dots for detection (Carrillo-Carrión et al., 2011). In the latter approach, eluate containing four separated purine bases (adenine, guanine, hypoxanthine, and xanthine) was mixed with Ag sol and passed through a Raman flow cell (Sheng et al., 1991). HPLC-SERS was also applied to the quantification of a folate antagonist methotrexate and its metabolites (Subaihi et al., 2017), amoxicillin in milk (Khrushchev et al., 2022), pesticides (Carrillo-Carrión et al., 2012; Wang et al., 2015), illicit drugs (Sägmüller et al., 2003; Trachta et al., 2004), etc. Also, the potential of on-line LC-SERS was demonstrated for untargeted tumor metabolomics. The method was applied to the determination of metabolites from cell lysate samples of tumors and showed results comparable with LC-MS (Xiao et al., 2020). SERS favorably compares to other detection methods due to the ability to identify certain class members of compounds owing to characteristic band patterns (Trachta et al., 2004).

#### 2.2.2 Gas chromatography

The coupling of gas chromatography with SERS detection is less common, mostly due to the advantages in mass spectrometric (MS) detection (Heaps and Griffiths, 2005), where the direct coupling of a GC with a mass spectrometer is easy to implement. Moreover, for GC-MS method, there exist mass spectral libraries, allowing the identification of compounds, which hinders the benefits of SERS. As a results, even though the combination of GC and SERS in principle is possible (Roth and Kiefer, 1994), the general acceptance is low (Heaps and Griffiths, 2005).

Several attempts were made to combine GC and SERS, e.g., trapping GC eluate either in liquid silver sol (“flow-cell” method) or on TLC plates coated with silver colloidal solution (“mobile-phase elimination” method) followed by SERS quantification of pyridine (Roth and Kiefer, 1994). Alternatively, an off-line approach was proposed for the quantification of caffeine and p-nitrothiophenol by condensing the analytes on a liquid-nitrogen-cooled silver surface that had been formed on the surface of a ZnSe plate by physical vapor deposition (Heaps and Griffiths, 2005).

## 3 Future perspectives

Even though a huge progress in coupling chromatographic separation with SERS detection has been achieved, there still exist opportunities and challenges in the application of these techniques, especially in substrate stability and reproducibility of results. Noble metal nanoparticles are widely used as SERS substrates; however, the sensitivity of detection is largely dependent on nano-substrate properties. To achieve more accurate and sensitive detection, the shape and formation of the nanoparticles of noble metals should be improved further (Zhang et al., 2017). To improve sensitivity and reproducibility of results, the development of SERS-active surfaces seems to be perspective. For obtaining higher accuracy, there is a great potential of applying machine-learning techniques, especially in the case of coeluting compounds or interferences from matrix components. Also, the application of noble metals, e.g., gold, silver, as SERS substrates hinders biocompatibility and poses other issues (Han et al., 2023). Another important aspect aimed at improving biocompatibility is reusability of the developed SERS-active surfaces. Since highly sensitive and accurate quantification of analytes in diverse complex matrices is required, there is a need to the reveal the potential of the SERS combined techniques on a wider range of target analytes and matrices. Preferably, the developed techniques should be simple, convenient, and inexpensive.
